# Outbreaks of epidemic keratoconjunctivitis caused by human adenovirus type 8 in the Tibet Autonomous Region of China in 2016

**DOI:** 10.1371/journal.pone.0185048

**Published:** 2017-09-15

**Authors:** Zhenqiang Lei, Zhen Zhu, Bai ma ci wang, Hong mei, Hong Li, Dan zeng gong ga, Guo jie, Mi ma bu chi, Sheng Zhang, Chaofeng Ma, Wenbo Xu

**Affiliations:** 1 School of Public Health, Shaanxi University of Chinese Medicine, Xianyang city, Shaanxi province, People’s Republic of China; 2 WHO WPRO Regional Reference Measles/Rubella laboratory and Key Laboratory of Medical Virology Ministry of Health, National Institute for Viral Disease Control and Prevention, Chinese Center for Disease Control and Prevention, Beijing City, People’s Republic of China; 3 Department of Infectious Diseases, Tibet Center for Disease Control and Prevention, Lhasa city, Tibet Autonomous Region, People’s Republic of China; 4 Department of Health Inspection, Tibet Center for Disease Control and Prevention, Lhasa city, Tibet Autonomous Region, People’s Republic of China; 5 Department of Pathogenic Biology, Medical School, Anhui University of Science and Technology, Huainan City, Anhui Province, People’s Republic of China; 6 Gongbujiangda County Center for Disease Control and Prevention, Linzhi City, Tibet Autonomous Region, People’s Republic of China; 7 Department of Infectious Diseases, Rikaze Prefecture Center for Disease Control and Prevention, Rikaze City, Tibet Autonomous Region, People’s Republic of China; 8 Xi'an Center for Disease Control and Prevention, Xi’an City, Shaanxi Province, People’s Republic of China; University of Hong Kong, HONG KONG

## Abstract

From April to November 2016, two outbreaks of epidemic keratoconjunctivitis (EKC) occurred successively at primary and middle schools in the Tibet Autonomous Region of China, and a total of 197 clinically diagnosed cases were reported. Real-time PCR analyses confirmed that human adenovirus (HAdV) infection was related to these outbreaks. Further studies involving sequence determination and phylogenetic analysis based on the penton base, hexon, and fiber genes indicated that human adenovirus type 8 (HAdV-8), belonging to species D, was responsible for the outbreaks. This is the first report of a HAdV-8 associated EKC outbreak in mainland of China, and the results of this study are expected to provide support for future research into HAdV-8 in China.

## Introduction

Human adenovirus (HAdV) belongs to the genus *Mastadenovirus* within the family *Adenoviridae*, and is a double-stranded DNA virus with non-enveloped and icosahedral capsids [1]. The HAdV capsid is composed of 252 capsomeres, which form three viral capsid proteins, namely penton base, hexon, and fiber [2]. Among them, the hexon and fiber proteins contain most of the neutralizing epitopes [3], and fiber provide attachment of virus with host cell and also responsible for agglutination of red blood cell [4]. On the basis of biological properties, a serum neutralization assay, and whole-genome sequencing, HAdV can be divided into seven species (A to G) and is further formally recognized as having 84 different types (http://hadvwg.gmu.edu/). Since the type-specific domains are mapped to Loop1 (HVR1–6) [5] and Loop2 (HVR7) [6] within the hexon gene, sequencing of any of two loops regions can be used for preliminary molecular typing of HAdV. However, owing to the recent emergence of recombinant HAdVs, sequencing and analysis of at least penton base, hexon, and fiber gene is required for more accurate HAdV typing (URL: http://hadvwg.gmu.edu).

Each type of HAdV is kno**w**n to have a unique and specific tissue tropism, and may infect respiratory tract [7, 8], intestines [9], eyes [10] or urinary tract [11], which then leads to a variety of clinical diseases. Epidemic keratoconjunctivitis (EKC) is a highly contagious and serious eye disease that usually occurs as an outbreak in schools, military camps, swimming pools, and other community settings [12, 13]. EKC is reportedly associated with adenoviruses (HAdV-8, 19, 37) and enteroviruses (EV-D70 and CV-A24) [14], among which, HAdV-8, belonging to species D, is the predominant causative agent, and accounts for up to 80% of EKC cases [15].

EKC was first described in 1889, and HAdV-8 was first identified as the causative agent of EKC (strain Trim, HAdV-8P) in the USA in 1955, and was subsequently isolated [15]. The next EKC outbreak was reported in Ghana, Africa in 1969 [16], after which HAdV-8 spread rapidly all over the world, causing severe and highly contagious eye diseases [17, 18]. A study into the seroprevalence of HAdV-8 showed that HAdV-8 infection was common in China’s neighboring countries or regions, such as Japan and Taiwan China [15]; however, an effective HAdV surveillance system had not been established in China, and therefore the epidemic background of HAdV-8 here was still unknown.

The current study identified HAdV-8 as the etiological agent in two EKC outbreaks that occurred in the Tibet Autonomous Region of China in 2016. This represents the first report of an HAdV-8-associated EKC outbreak in mainland of China. The results of this study are expected to provide support for future research into HAdV-8 in China.

## Materials and methods

### Ethical statement

This study was approved by the second session of Ethics Review Committee of the National Institute for Viral Disease Control and Prevention (IVDC) at China CDC. All methods were performed in accordance with the relevant guidelines and regulations. Written informed consent was obtained from all participants or legal guardians involved in this study for the collection of conjunctival swab specimens or throat swabs for pathogenic identification of EKC outbreaks.

### Specimen collection

During the first EKC outbreak in Gongbujiangda County, a total of 41 conjunctival swab specimens were collected from 44 clinically confirmed EKC patients from four schools during the acute phase of infection, by epidemiology staff at the Gongbujiangda Centers for Disease Control and Prevention (CDC). During the second EKC outbreak in Namling County, six throat swabs and six conjunctival swabs were collected from six patients within 3 d from the date of disease onset, by epidemiology staff at of Namling CDC.

After aseptic collection, specimens were transferred into 2 ml viral transport medium (Kindu co., Shanghai China). Specimens were stored at 4°C and delivered to the IVDC at the China CDC within 24 h, under the cold chain, for further investigation.

### Viral nucleic acid extraction, identification, and preliminary typing

Viral nucleic acids were extracted directly from clinical specimens from the EKC cases using a QIAamp Viral RNA Mini Kit (Qiagen, Valencia, CA) and QIAamp DNA Mini Kit (Qiagen, Valencia, CA), according to the manufacturer’s instructions. Real-time reverse transcription-polymerase chain reaction (rRT-PCR) and real-time PCR (rPCR) were performed using a One Step PrimeScript^™^ RT-PCR Kit (Perfect Real Time; TaKaRa Biotechnology (Dalian) Co., Ltd.). Specific degenerate primers and probes used to identify the broad spectrum of human enterovirus (HEV) [19] and HAdV infection, respectively, are listed in Table 1. The 194-bp and 96-bp amplicons were generated and detected using TaqMan probes for HEV and HAdV, respectively, both of which were labeled with carboxyfluorescein (FAM) and Black Hole Quencher dye. Amplification was performed using an ABI 7500 Real-Time PCR system (Life Technologies, NY, USA) and data were analyzed using ABI 7500 SDS software (version 2.0.1). The HAdV-positive samples used to amplify the Loop2 region within the hexon gene for preliminary molecular typing of HAdV were as previously reported [6]. To exclude possible cross contamination, negative controls for extraction and rPCR steps were included in each test.

**Table 1:** Sequences of primers and probes for rPCR used to identify broad spectrum HEV and HAdV.

Virus	Type	Name	Sequence (5′-3′ orientation)	Position
EV	Primer	EV(YG)F	GGCTGCGYTGGCGGCC	361–376^a^
	Primer	EV(YG)R	CCAAAGTAGTCGGTTCCGC	536–554^a^
	Probe	EVTY(YG)PB	FAM-CTCCGGCCCCTGAATGCGG-BHQ1	450–468^a^
HAdV	Primer	HAdV-F	GGACGCCTCGGAGTACCTGAG	18472–18492^b^
	Primer	HAdV-R	ACIGTGGGGTTTCTGAACTTGTT	18545–18567^b^
	Probe	HAdV-P	FAM-CTGGTGCAGTTCGCCCGTGCCA-BHQ1	18500–18521^b^

^a^Sequence position of primers and probe referring to HEV71 (BrCr strain, GenBank accession number U22521).

^b^Sequence position of primers and probe referring to HAdV-7(Strain Gomen, GenBank accession number AY594255).

### Cell culture and virus isolation

Virus isolation from HAdV-positive samples was performed using HEp-2 cells obtained from the American Type Culture Collection (ATCC Number CCL-23), according to standard procedures [8]. Cytopathic effect (CPE) was observed daily, and the virus was harvested when > 70% cells had developed adenovirus-like CPE, such as rounding and swelling of cells, followed by detachment from the culture surface into grape-like clusters [15] within 7 d. If CPE was not observed within 7 d of incubation, two extra passages were performed.

### PCR amplification and sequencing of penton base, hexon, and fiber genes

Viral DNA was extracted from the cultured virus suspension using a QIAamp DNA Mini Kit (Qiagen, Valencia, CA, USA), according to the manufacturer's instructions. To further identify the HAdV type and exclude potential recombination events, PCR was performed to specifically amplify penton base, hexon, and fiber genes, using a Platinum PCR SuperMix (Invitrogen, Carlsbad, CA, USA). Primer pairs are listed in Table 2. Primers used for PCR amplification were designed based on the HAdV-8 sequence (Trim strain; GenBank accession number AB448767), using a PCR primer design tool (Primer 3, Version 2.3.7). PCR products were purified using a QIAgel Extraction Kit (Qiagen, Valencia, CA, USA), and sequenced using Sanger sequencing with BigDye Terminator chemistry (Version 3.1; Life Technologies, NY, USA) and a 3130 Genetic Analyzer (Life Technologies, Japan).

**Table 2:** Primers used for amplification and sequencing of the penton base, hexon, and fiber genes of HAdV-8.

Primer	Sequence (5′-3′ orientation)	Position^a^
HAdV8-penton-F	ATAGCAGCGTGTTGGACTTG	13385–13404
HAdV8-penton-R	TTCTTGGCTCCTCCGTACAT	15150–15169
HAdV8-hexon-1F	GTATCGTGGGCCTAGGAGTG	17659–17678
HAdV8-hexon-1R	GCCCGTTCATGTACTCGTAG	19245–19364
HAdV8-hexon-2F	GTGGCTGCTCAAAACCAAAT	19081–19100
HAdV8-hexon-2R	GTGGGTGCCCAAAAAGTAGG	20665–20684
HAdV8-fiber-F	CTCCCAGCTCTGGTACTCCA	30680–30699
HAdV8-fiber-R	TGGTGGTGGGAGAATGTGTA	31942–31961

^a^Nucleotide positions are indicated according to prototype HAdV-8 strain (strain Trim; GenBank accession number AB448767).

### Sequence analysis

Editing and splicing of sequence data were performed using Sequencher software (Version 5.4.5, Gene Codes Corporation, USA). Basic Local Alignment Search Tool (BLASTn program; National Center for Biotechnology Information, Bethesda, MD, USA) was used to identify homologous nucleotide sequences in the GenBank database. Sequence alignment and phylogenetic trees were created using MEGA software (Version 5.0.3) [20]. Phylogenies were constructed using a maximum likelihood method with Tamura-Nei model; other parameters in the MEGA software were set to default.

### Nucleotide sequence accession numbers

Nucleotide sequences of the entire penton base, hexon, and fiber genes from two representative HAdV-8 strains from the two outbreaks were deposited in the GenBank database with accession numbers as follows: strain human/CHN/Tibet/05-12/2016/8 (penton base, MF363095; fiber, MF363097; hexon, MF363099); strain human/CHN/Tibet/10-03/2016/8 (penton base, MF363094; fiber, MF363096; hexon, MF363098).

## Results

### Epidemiology analysis

The first EKC outbreak began on April 10, 2016, with a student from a primary school (School 1) in Gongbujiangda County of Linzhi city in the Tibet Autonomous Region of China. The student experienced bilateral conjunctival congestion, photophobia, and lacrimation. The symptoms were not alleviated by treatment with antibiotic eye drops. However, after the student was treated with combined antibiotic and antiviral (acyclovir eye drops) therapy, the symptoms did gradually ease, and eventually recover over the next 7 d. Subsequently, some other students from the same primary school presented with photophobia, unilateral or bilateral conjunctival mild hyperemia, blurred vision, and foreign body sensation, which didn’t occur among these students before. From April 17, 2016, the epidemic gradually spread to three other schools within Gongbujiangda County, including two primary schools (School 2 and School 3) and one middle school (School 4). The farthest distance between the schools was about 64 kilometers. By May 5, 2016, a total of 44 students (ranging in age from 7 to 14 years) were affected by this outbreak (10 cases at School 1, 23 cases at School 2, 4 cases at School 3, and 7 cases at School 4).

Five months later, a second EKC outbreak occurred in Namling County of Rikaze city, which is about 560 kilometers away from Gongbujiangda County of Linzhi city. The index case, a male student from No.1 Middle School in Namling County of Rikaze city, was diagnosed as a suspected EKC case by the health center and reported to Namling CDC on October 5, 2016. This patient had no history of contact with EKC cases. The number of cases in the school subsequently increased, and by November 23, 2016, 153 suspected cases (ranging in age from 13 to 17 years) were identified among the 1603 students at the school. Through symptomatic treatment and combined antibiotic and antiviral therapy, all patients recovered without complications. No additional cases of infection were reported at any other schools in Namling County.

### Pathogenic identification of the EKC outbreaks

Viral nucleic acids were extracted directly from 53 clinical specimens, including 41 conjunctival swab specimens collected during the first outbreak in Gongbujiangda County, and six throat swabs and six conjunctival swabs collected from the second outbreak in Namling County. rPCR and rRT-PCR were performed using primer pairs and probes specific to HAdV and HEV [19], respectively, and HAdV was detected only in positive samples. Of all the specimens analyzed, 38/41 from the first outbreak and 12/12 from the second outbreak were identified as HAdV-positive. Our focus then shifted to identifying the type of HAdV present in each sample.

For preliminary type identification, all HAdV-positive samples were positive for the partial hexon gene containing Loop2 region (nt18748 to 19367, Trim strain, GenBank accession number AB448767). Twenty of the 38 HAdV-positive samples from the first outbreak and 10 of the 12 HAdV-positive samples from the second outbreak were successfully amplified, and the sequences of the partial hexon gene from all of the samples were identical. BLAST sequence analysis revealed that all sequences had the highest grade of homology (100%) with HAdV-8 strains in the GenBank database.

**Fig 1:**
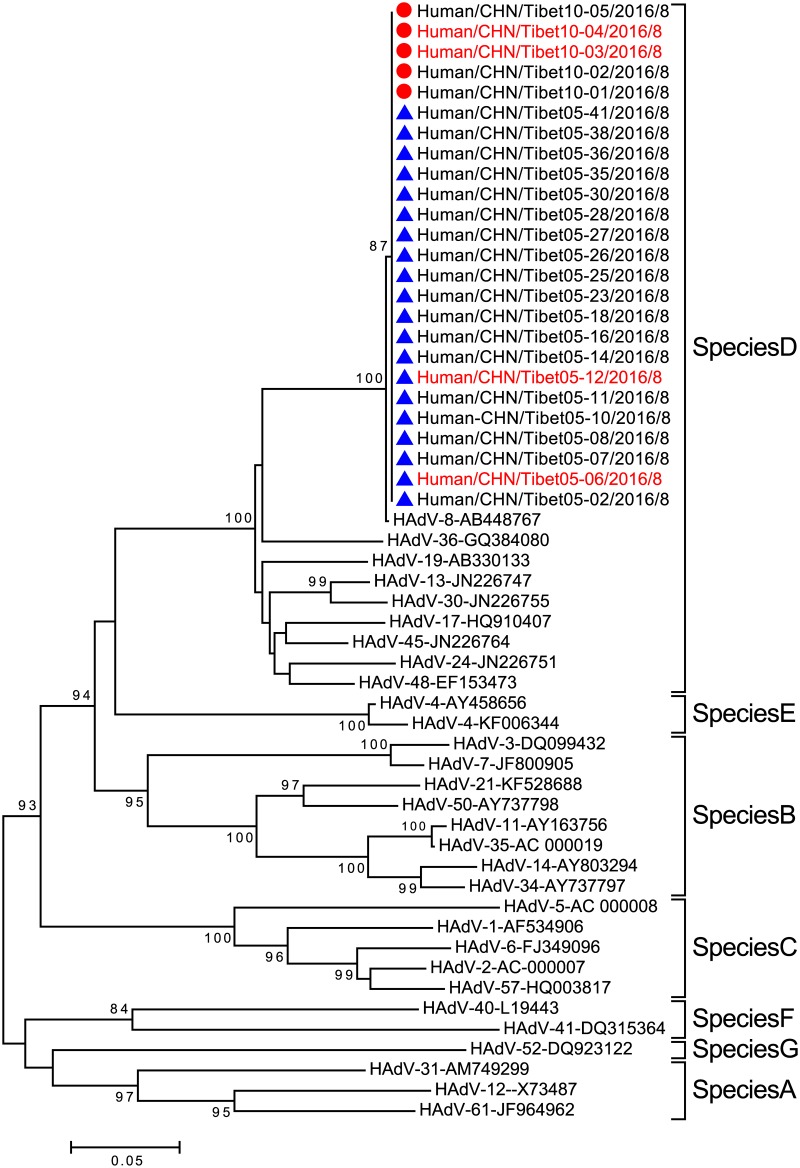
Preliminary type identification of samples collected from two EKC outbreaks in Tibet in 2016, based on the partial hexon gene. Phylogenetic trees were generated using the maximum-likelihood method with 1000 replicates. Samples collected in Namling County are indicated by red dots and samples from Gongbujiangda County are indicated by blue triangles. Strains shown in red were used for further analysis. Sequences of the 30 prototype HAdV strains representing the seven HAdV species (A-G) are available in the GenBank database.

A phylogenetic analysis was then conducted, based on the partial hexon gene with sequences from 30 samples from the EKC outbreaks and 30 prototype HAdV strains representing the seven HAdV species (A-G) (Fig 1). The sequences of all samples from the two outbreaks clustered with HAdV-8 within species D of HAdV, which was supported by high bootstrap values. Therefore, the results of the BLAST sequence analysis together with the phylogenetic analysis indicate that all patients from the two EKC outbreaks in Tibet were infected with the same virus, which belonged to HAdV-8 within species D of HAdV.

### Phylogenetic analysis of penton base, hexon, and fiber genes

After rPCR identification, all HAdV-positive samples were inoculated into HEp-2 cells and cultured at 37°C for 7 d. A total of 30 HAdV isolates were obtained (20 from the first outbreak and 10 from the second), and the penton base, hexon, and fiber genes were successfully amplified by PCR from all isolates using HAdV-8-specific primers to obtain the predicted products of 1563 bp (penton base), 2829 bp (hexon), and 1089 bp (fiber) (Table 2). Sequence analysis revealed that the 30 HAdV strains exhibited 100% nucleotide sequence similarity in all three genes, providing further evidence that all patients were infected with the same virus. Two representative strains from each outbreak (Human/CHN/Tibet10-03/2016/8, Human/CHN/Tibet10-04/2016/8, Human/CHN/Tibet05-06/2016/8, Human/CHN/Tibet05-12/2016/8) were selected for phylogenetic analysis.

**Fig 2:**
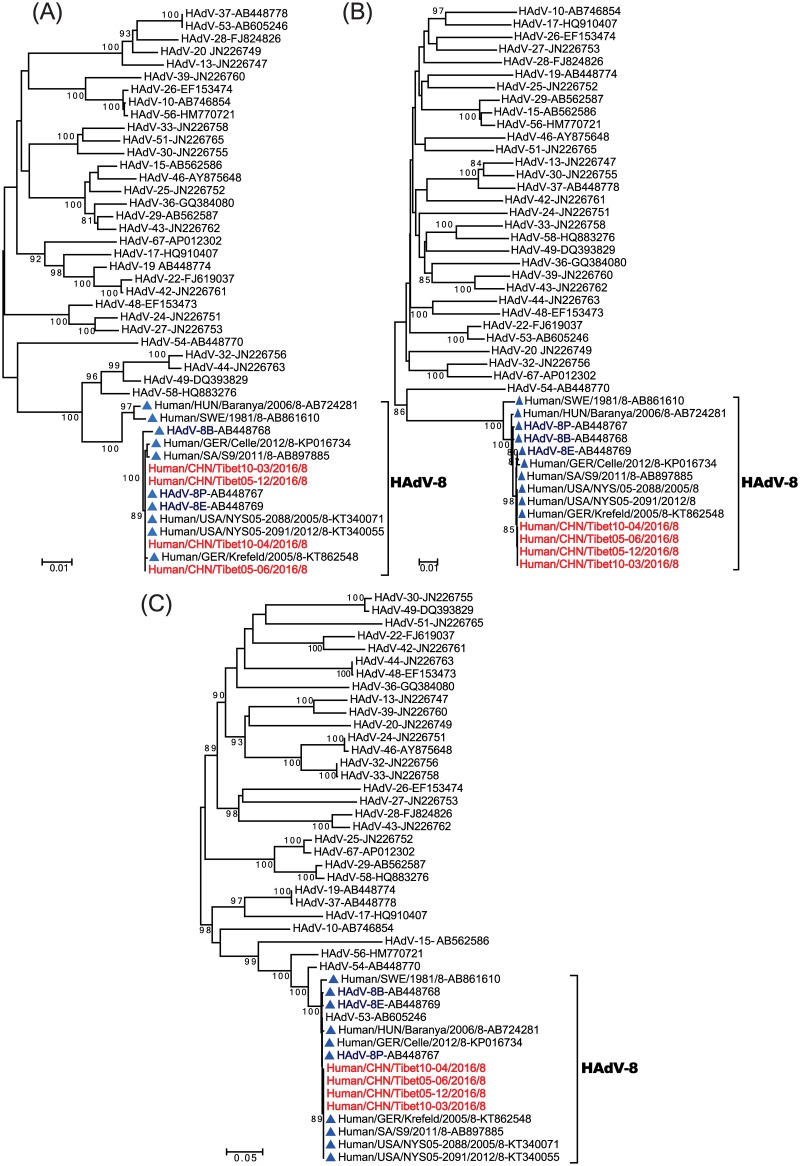
Phylogenetic analysis of sequences of HAdV-8 strains from the outbreaks in Tibet compared to HAdV-8 strains from other countries and 32 HAdV species D viruses based on (a) the entire penton gene; (b) the entire hexon gene; (c) the entire fiber gene. Phylogenetic trees were generated using the maximum-likelihood method with 1000 replicates. HAdV-8 strains isolated in Tibet are highlighted in red, and HAdV-8 strains from other countries are indicated by blue triangles.

To investigate genetic relationships between the Tibet HAdV-8 strains and other strains within species D of HAdV, phylogenetic analyses were conducted based on the entire penton base, hexon, and fiber genes, using 32 known virus strains of HAdV species D (Fig 2). The results revealed that all of the Tibet HAdV-8 strains segregated into monophyletic clusters within HAdV-8.

To our knowledge, this is the first report of an HAdV-8 outbreak in China. Therefore, we compared the Tibet HAdV-8 strain with known global HAdV-8 strains, and constructed a phylogenetic tree (Fig 2). Ten HAdV-8 strains identified from 6 countries between 1955 and 2012, including Hungary (1 strain), Sweden (1 strain), Japan (2 strains) [17], Germany (2 strains), Saudi Arabia (1 strain), and the United States (3 strains) were selected for this analysis. The Tibet HAdV-8 strains showed high nucleotide identity with the global strains. In the penton base, hexon, and fiber genes, the nucleotide and amino acid identities between the Tibet HAdV-8 strains and HAdV strains from other countries were about 98.2–100% with 0–27 nucleotide substitutions and 99.2–100% with 0–4 amino acid substitutions (penton base), 98.5–99.9% with 1–40 nucleotide substitutions and 99.2–100% with 0–7 amino acid substitutions (hexon), and 98.9–100% with 0–11 nucleotide substitutions and 98.6–100% with 0–5 amino acid substitutions (fiber). Among the 10 global HAdV-8 strains, the Tibet strain was most different from the Sweden strain isolated in 1981 (strain Human/SWE/1981/8), and least different from the strains from Germany (2005), Saudi Arabia (2011), and the United States (2005, 2012) for all three of the genes. The sequence of the Tibet strain was also similar to HAdV-8P [15], HAdV-8B [15], and HAdV-8E [15] (< 0.4% nucleotide difference), based on all three genes. All these results indicate that the HAdV-8 genome is highly conserved from the sequencing of the three genes.

## Discussion

EKC is a severe infectious eye disease frequently associated with HAdV-8, which has been isolated from both epidemic and sporadic cases from many different countries and regions [17]. In 2016, two EKC outbreaks occurred successively at primary and middle schools in the Tibet Autonomous Region of China. The results of the present study indicate that HAdV-8, which belongs to species D, was responsible for the outbreaks. Moreover, the viruses from the two EKC outbreaks were highly identical, suggesting that the outbreaks were caused by the same HAdV-8 strain.

The results of the current study revealed that HAdV-8 had been continuously circulating in Tibet for at least 8 months after the first case in April 2016. However, due to poor epidemiological investigation and the lack of HAdV surveillance, the origin and route of HAdV-8 transmission in Tibet remained unclear. Although the sequences from the two EKC outbreaks are identical, all the HAdV-8 genome is highly conserved from the sequencing of the three genes, and the two counties where the two EKC outbreaks occurred is about 560 kilometers away, so it is difficult to draw the conclusion that the EKC students from the two counties had any contact with each other. HAdVs are highly resistance to environmental conditions, and an epidemic could therefore last weeks or months [21, 22]. The timely identification of the pathogens in the present study was able to guide public health staff to take action against the outbreaks, and no more cases were reported after November 2016.

According to restriction endonuclease analysis, HAdV-8 strains can be divided into genome types HAdV-8A to 8K or HAdV-8/D1 to HAdV-8/D12, based on European and Asian classification criteria, respectively [15, 17]. Of these, HAdV-8P and HAdV-8E are thought to have worldwide distribution [15, 17]. Owing to the identification of new recombinant HAdVs in recent years, sequencing of three genes, penton base, hexon, and fiber, followed by phylogenetic analysis, is now recommended for HAdV type identification. This has replaced the neutralization test and restriction endonuclease analyses, which can lead to misclassification of strains [23–25]. In the present study, phylogenetic analysis indicated that the Tibet HAdV-8 strains had no recombination events in the main capsids, and showed very little genetic variation with global HAdV-8 strains, including HAdV-8P isolated in 1955. Thus, HAdV-8 has circulated worldwide but has displayed a relatively conserved and stable genome for almost 60 years, since its first isolation in 1955. These characteristics will make the selection of a vaccine strain for the virus much simpler in the future.

In addition to the common types of HAdV (HAdV-8,19, 37) [26], new HAdVs within HAdV-D, namely HAdV-53 [27], HAdV-54 [24], and HAdV-56 [28], were identified during adenoviral EKC epidemics in recent years. For example, an EKC outbreak associated with HAdV-56 was reported in Dalian city, China in 2012, in which a total of 451 EKC cases were identified [28]. EKC associated with HAdV-54 can lead to severe symptoms, including corneal opacity and has caused a nationwide epidemic in Japan since 2004 [29]. Therefore, EKC is a highly contagious disease, and etiological surveillance is extremely necessary.

In conclusion, this is the first report that details two EKC outbreaks caused by HAdV-8 in the Tibet Autonomous Region of China in 2016. Owing to insufficient surveillance, the circulation pattern of HAdV-8 in China remains unknown. Therefore, a nationwide epidemiological and virological surveillance of HAdVs in China must be established with some urgency, in order to understand the geographical distribution of HAdV types and provide some insight into disease control and prevention.
